# Accuracy of DXA in estimating body composition changes in elite athletes using a four compartment model as the reference method

**DOI:** 10.1186/1743-7075-7-22

**Published:** 2010-03-22

**Authors:** Diana A Santos, Analiza M Silva, Catarina N Matias, David A Fields, Steven B Heymsfield, Luís B Sardinha

**Affiliations:** 1 Exercise and Health Laboratory, Faculty of Human Movement-Technical University of Lisbon, Portugal; 2 Department of Pediatrics, Children's Medical Research Institute's Metabolic Research Program, University of Oklahoma Health Science Center, OK, USA; 3 Clinical Research, Metabolism, Merck Research Laboratories, Rahway, NJ, USA

## Abstract

**Background:**

Dual-energy x-ray absorptiometry (DXA) provides an affordable and practical assessment of multiple whole body and regional body composition. However, little information is available on the assessment of changes in body composition in top-level athletes using DXA. The present study aimed to assess the accuracy of DXA in tracking body composition changes (relative fat mass [%FM], absolute fat mass [FM], and fat-free mass [FFM]) of elite male judo athletes from a period of weight stability to prior to a competition, compared to a four compartment model (4C model), as the criterion method.

**Methods:**

A total of 27 elite male judo athletes (age, 22.2 ± 2.8 yrs) athletes were evaluated. Measures of body volume by air displacement plethysmography, bone mineral content assessed by DXA, and total-body water assessed by deuterium dilution were used in a 4C model. Statistical analyses included examination of the coefficient of determinant (r^2^), standard error of estimation (SEE), slope, intercept, and agreement between models.

**Results:**

At a group level analysis, changes in %FM, FM, and FFM estimates by DXA were not significantly different from those by the 4C model. Though the regression between DXA and the 4C model did not differ from the line of identity DXA %FM, FM, and FFM changes only explained 29%, 36%, and 38% of the 4C reference values, respectively. Individual results showed that the 95% limits of agreement were -3.7 to 5.3 for %FM, -2.6 to 3.7 for FM, and -3.7 to 2.7 for FFM. The relation between the difference and the mean of the methods indicated a significant trend for %FM and FM changes with DXA overestimating at the lower ends and underestimating at the upper ends of FM changes.

**Conclusions:**

Our data indicate that both at group and individual levels DXA did not present an expected accuracy in tracking changes in adiposity in elite male judo athletes.

## Background

In combat sports, athletes are subdivided into weight divisions. In order to qualify for their respective weight category, many athletes undergo a impressive weight changes preceding the competition [1]. With this drastic weight loss, lean body weight and percentage of fat mass (%FM) decrease [2,3]. Differences related to body mass, stature, and body composition may significantly influence fighting strategies (including technical and tactical skills) and consequently the physiologic profile of these athletes [4,5]. Thus, the ability of researchers and coaches to accurately estimate minimal changes in fat mass (FM) and fat-free-mass (FFM) is crucial in sports with weight categories (i.e., martial arts, wrestling, weight lifting, and combat sports) [6].

The lack of an easy, valid, and quick body composition method to assess FM and FFM does not allow the estimation of a correct minimal weight for athletes in specific sports, such as wrestling and the martial arts (e.g. judo and karate). At the most basic molecular level the human body is divided into two compartments, FM and FFM. Although FM is a relatively homogenous component of the body, FFM is an heterogeneous mixture of water, mineral, protein, and other minor constituents [7]. Many body composition methods assume that some of these components have a stable proportion within the FFM, and therefore a constant value of 1.1 g/cc is used for the fat-free mass density (FFM_D_) [7,8]. However, assuming that FFM_D _is stable increases the error in fat mass estimation as interindividual differences might not be considered, particularly in specific groups such as athletes [9-11]. This means the accuracy in the estimation of body composition increases when methods rely on fewer assumptions of the components of the FFM [12].

A four-compartment (4C) model using the updated predicted model for soft-tissue minerals developed by Wang and colleagues [13], is an example of a criterion method for assessing FM, as the major FFM molecular components are estimated [total body water (TBW), mineral and protein] and less assumptions are made for determining FM [14]. Despite their advantages, few studies have used 4C models to evaluate changes in body composition in athletes [15]. The elevated costs implicated in assessing all the body components required for the use of this criterion model, and the lack of accessibility may justify the limited number of follow-up body composition studies in the literature, especially in athletes. Thus, other less expensive alternative methods need to be validated for tracking body composition in athletes. Dual-energy X-ray absorptiometry (DXA) seems to present an excellent alternative to 4C models as the systems are affordable, practical, require no subject involvement, and impose minimal risk [16]. DXA permits quantification of multiple whole body and regional components, including bone mineral (Mo), fat, and lean soft tissue (LST) [17,18]. Although DXA has been validated in cross-sectional analysis in athletes [12], it has not been validated for estimating changes in %FM, FM and FFM in combat sports in general and Judo athletes in particular. Thus, the purpose of the present study was to examine the accuracy of DXA in tracking body composition of elite male Portuguese judo athletes from a period of stability to prior to a competition, comparing with a 4C criterion.

## Methods

### Subjects

Twenty-seven male judo athletes were eligible to participate in the study. The subject inclusion criteria were: 1) age ≥ 18 years, 2) practiced rapid volitional weight reduction greater than three times in the past year, 3) ≥ of 5 years of experience, 4) ≥ 15 hours training per week; 5) minimum technical level of 1^st ^degree black belt, 6) negative test outcomes for performance enhancing drugs; and 7) not taking any medications or dietary supplements. Medical screening indicated that all subjects were in good health, without endocrine abnormalities that would limit their participation in the study. All subjects were informed about the possible risks of the investigation before giving their written informed consent to participate; all procedures were approved by the Institutional Review Board of the Faculty of Human Movement, Technical University of Lisbon.

### Experimental Design

A convenience sample of national top-level Judo athletes, engaged on this sport for more than 7 years, was used. Data collection was performed between September (1 month after the beginning of the in-season) to December.

Body composition assessment was made during a period of weight stability and again prior to competition. A period of about one month was used between the period of stability and prior to competition. The period of weight stability is considered the baseline phase with judo athletes performing their regular regimens of judo training which typically last ~2 h in the morning and ~2 h in the evening. Two of the morning sessions were used for improving cardiorespiratory fitness and strength while the other sessions consisted of judo specific skills and drills and randori (fighting practice) with varying intensity above and up to 90-95% of maximal oxygen consumption (VO_2_max). Prior to competition some of these athletes lost weight through energy and/or fluid restriction while others remained or increased their body weight.

### Body Composition Measurements

Subjects came to the laboratory at the period of stability and prior to competition, after a 12-hour fast, and refrained from exercise for at least 15-hours, alcohol or stimulant beverages.

All subjects were informed about the research design and signed a consent form according to the regulations of the Ethical Committee of the Faculty of Human Movement, Technical University of Lisbon.

All measurements were carried out in the same morning. In brief, the procedures are described as follows:

#### Anthropometry

Subjects were weighed to the nearest 0.01 kg wearing a bathing suit without shoes on an electronic scale connected to the plethysmograph computer (BOD POD^©^, Life Measurement, Inc., Concord, CA, USA). Height was measured to the nearest 0.1 cm with a stadiometer (Seca, Hamburg, Germany), according to the standardized procedures described elsewhere [19].

#### Hydration Status

To assure all athletes were in a neutral hydration state at the period of stability, we observed if voided urine was pale yellow (i.e., dilute) and we confirmed with the athlete that post-voiding first-morning body weights for the 3 days prior the first visit did not change by over 1% [20].

#### Dual-Energy X-ray Absorptiometry

To estimate FM and FFM, DXA measurements were made with a total body scan (QDR 4500A, fan-beam densitometer, software version 8.21; Hologic, Waltham, USA) that measured the attenuation of X-rays pulsed between 70 and 140 kV synchronously with the line frequency for each pixel of the scanned image. Following the protocol for DXA described by the manufacturer, a step phantom with six fields of acrylic and aluminum of varying thickness and known absorptive properties was scanned alongside each subject to serve as an external standard for the analysis of different tissue components. The same laboratory technician positioned the subjects, performed the scans and executed the analysis according to the operator's manual using the standard analysis protocol. Based on ten subjects, the coefficient of variation (CV) in our laboratory for FM and FFM were 2.9 and 1.7%, respectively.

#### Four-Compartment Model

A four-compartment model was used as the reference method, calculated after using the total-body soft tissue mineral (Ms) component obtained as Ms = 0.0129 × TBW [13]. The model is described as follow:(1)

Where BV is body volume (L), TBW is total body water (kg), Mo is bone mineral (kg), Ms is total-body soft tissue mineral (kg) and BW is body weight (kg).

Accordingly, equation 1 was then recalculated as:(2)

Where BV is body volume (L), TBW is total body water (kg), Mo is bone mineral (kg), and BW is body weight (kg).

Total body mineral (M) was calculated as(3)

Where Mo is bone mineral (kg), and Ms is total-body soft tissue mineral (kg)

### Calculation of Density of Fat-free Mass

The FFM_D _was estimated from TBW, Mo, Ms and protein (protein is equal to BW minus FM from the 4C model, TBW, Mo and Ms) contents of FFM (estimated as BW minus FM from the 4C model) and their densities (0.9937, 2.982, 3.317 and 1.34 g/cc), for TBW, Mo, Ms and protein, respectively,(4)

Where _D _is density, FFM is fat-free mass; TBW is total body water; Mo is bone mineral, and Ms is total-body soft tissue mineral.

#### Bone Mineral

DXA, a Hologic model QDR 4500A fan-beam densitometer (QDR-4500, Hologic, Waltham, USA), was used to measure bone mineral content (BMC) by using a software version 8.21. Scan positioning, acquisition, and analysis were standardized. All subjects had fan-beam scans. Considering that BMC represents ashed bone, BMC was converted to total-body bone mineral (Mo) by multiplying it by 1.0436 [21]. Based on test-retest using 10 subjects, the total error of measurement (TEM) and the CV for BMC in our laboratory were 0.02 kg, and 1.6%, respectively.

#### Body Volume

Body volume (BV) was assessed by air displacement plethysmograph (ADP) (Life Measurement, Inc., Concord, CA, USA). The use of this method is described in detail elsewhere [22,23]. Briefly, after voiding the bladder, each subject was weighed to the nearest gram while wearing a swimsuit. The ADP device was calibrated according to the manufacturer's instructions, and raw body volume (Vbraw) was determined. The effects of clothing and hair are accounted for by using minimal clothing, such as a bathing suit, and by compressing hair with a swim cap. Finally, thoracic gas volume (Vtg) was measured in the BOD POD^® ^by using a technique, common to standard pulmonary plethysmography, called the "panting maneuver". While wearing a nose clip, the subjects breathed through a tube; after two to three normal breaths, the airway occluded for 3s at mid-exhalation. During this time, the subject was instructed to gently puff against the occlusion by alternately contracting and relaxing the diaphragm. At the period of stability Vtg was measured in all subjects and was entered during their one month follow-up pre competition assessment.

All measurements were conducted with software version 1.68. The TEM and CV for BV, based on test-retest using 10 subjects, were 0.2 L, and 0.5%, respectively.

#### Total Body Water

Total-body water was assessed by the deuterium dilution technique using a stable Hydra gas isotope ratio mass spectrometer (PDZ, Europa Scientific, UK). After a completed 12 h fast, an initial urine sample was collected and a deuterium oxide solution dose (2H_2_O) of 0.1 g/kg of body weight diluted in 30 ml of water was immediately administered. After a 4 h equilibration period, a new urine sample was collected. The amount of 2H_2_O in the isotope dilutions was analyzed. Urine and diluted dose samples were prepared for 1H/2H analysis using the equilibration technique of Prosser and Scrimgeour [24]. After the tubes were filled they were equilibrated at 20 ± 1°C overnight for 3 days. The tubes were then introduced sequentially into a helium flow that was dried by magnesium perchlorate, and then analyzed by a Hydra gas isotope ratio mass spectrometer set to detect 1H/2H. The enrichments of equilibrated local water standards were calibrated against standard mean ocean water (SMOW). Based on delta SMOW, TBW was estimated by including a 4% correction due to the recognized amount corresponding to deuterium dilution in other compartments [25]. The TEM and CV for TBW with the stable isotope ratio mass spectrometry in this laboratory were 0.3 kg and 1.3%, respectively.

#### Propagation Measurement Error

In the present study we selected ADP to assess BV, DXA to estimate Mo, and the deuterium dilution technique to estimate TBW. The propagation of measurement errors associated with the determination of BV, TBW, and Mo can be calculated by assuming that the squared technical errors of measurement (TEM^2^) are independent and additive [26]. Accordingly,(5)

using the above equation,(6)

The test-retest reliability data collected in the present study thus yields a value of ~1%FM units.

### Statistical analysis

Data were analysed with PASW Statistics for windows version 18.0, 2009 (SPSS Inc., an IBM Company, Chicago). Descriptive statistics including means ± SD were calculated for all outcome measurements.

Using 27 subjects, this study is 80% powered to detect a correlation coefficient higher than 0.51 or lower than -0.51. Comparison of group means was performed using paired sample t-Test and wilcoxon test when normality was not verified. One sample t-Test was used to compare group means with reference population. Simple linear regressions were performed to calculate the relationship between FM, %FM, and FFM estimated by the reference 4C model and DXA. Agreement between methods was assessed [27], including the 95% limits of agreement. The correlation between the mean of the reference method and the assessed method with difference between both was used as an indication of trend. Also, correlation between the differences of the methods and potential variables were performed. For all tests, statistical significance was set at p < 0.05.

## Results

Subject demographic data is presented in Table 1.

**Table 1:** Subject characteristics at the period of stability, prior to competition, and respective changes.

N = 27	Period of Stability	Prior to Competition	Changes
	
	Mean ± SD	Mean ± SD	Mean ± SD
Age (yrs)	22.2 ± 2.8		

Stature (m)	1.76 ± 0.05		

Weight (kg)	72.8 ± 7.1	72.0 ± 6.9	-0.87 ± 1.93

BMI (kg/m^2^)	23.6 ± 2.3	23.4 ± 2.2	-0.28 ± 0.64

Abbreviations: BMI: body mass index;

*Changes are calculated as: prior to competition minus period of stability

The mean and SDs at the period of stability, prior to competition, and respective differences in %FM, FM, and FFM are summarized in table 2.

**Table 2:** Body composition variables at the period of stability, prior to competition, and respective differences

N = 27	Period of Stability	Prior to Competition	Changes
	
	Mean ± SD	Mean ± SD	Mean ± SD
%FM_4C_	9.2 ± 4.1	8.0 ± 3.8	-1.22 ± 2.70^b^

FM_4C _(kg)	6.8 ± 3.3	5.9 ± 3.0	-0.94 ± 1.98^b^

FFM_4C_(kg)	66.1 ± 6.4	66.1 ± 6.0	0.07 ± 2.04

%FM_DXA_	12.1 ± 3.1^b^	11.7 ± 2.8^b^	-0.41 ± 1.05

FM_DXA _(kg)	8.8 ± 2.8^b^	8.4 ± 2.5^b^	-0.42 ± 0.93^a^

FFM_DXA _(kg)	63.4 ± 5.7^b^	62.9 ± 5.8^b^	-0.45 ± 1.55

FFM_D _(g/cc)	1.100 ± 0.007	1.102 ± 0.009	0.002 ± 0.005

TBW/FFM	0.72 ± 0.02^c^	0.71 ± 0.03^c^	0.006 ± 0.016

M/FFM	0.057 ± 0.003^c^	0.057 ± 0.004^c^	0.000 ± 0.002

Protein/FFM	0.22 ± 0.02^c^	0.23 ± 0.03^c^	0.006 ± 0.016

Abbreviations: %FM: relative fat mass; FM: fat mass; FFM: fat-free mass; 4C: four-compartment model; DXA: dual-energy x-ray absorptiometry; FFM_D_: density of fat-free mass; TBW/FFM: fat-free mass hydration; M/FFM: mineral fraction of fat-free mass; Protein/FFM: protein fraction of fat-free mass

*Changes are calculated as: prior to competition minus period of stability

^a ^Significant difference from the period of stability and prior to competition, p < 0.05

^b ^Significant difference from the reference method, p < 0.05;

^c ^Significant difference from the value obtained based on chemical cadaver analysis, p < 0.05 (FFM_D _= 1.1 g/cc [7]; TBW/FFM = 0.738; M/FFM = 0.068; Protein/FFM = 0.194)

DXA significantly overestimated %FM and FM, and underestimated FFM in relation to the 4C model in cross-sectional analysis (p < 0.05), although no differences between methods were observed when tracking changes (p > 0.05).

**Figure 1:**
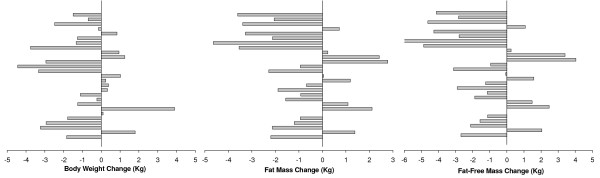
**Individual body composition changes assessed by the 4C model**. Individual body weight, absolute FM, and FFM changes (calculated as prior to competition minus period of stability).

Individual results showed that %FM, FM, FFM and weight changes ranged from -7.00 to 4.04%, from -4.64 to 2.78 kg, from -5.72 to 4.10 kg, and from -4.44 to 3.90, respectively (figure 1). Individual body weight changes of less than 1 kg were found in 6 athletes while in FM and FFM 8 and 12 athletes, respectively, presented changes of less than 1 kg.

**Figure 2:**
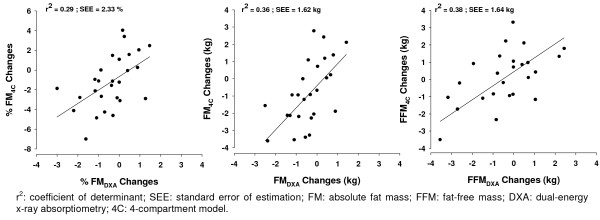
**Regression between methods in tracking body composition**. Regression of %FM, FM and FFM changes (calculated as prior to competition minus period of stability) obtained by DXA with the 4C. All the regression lines, did not differ from the line of identity, as the slope and intercept were not different from 1 and 0, respectively (p > 0.05).

The body composition changes obtained using DXA were related with those obtained from the 4C model in tracking %FM (r = 0.53, p = 0.004), FM (r = 0.60, p = 0.001) and FFM (r = 0.62, p = 0.001). The accuracy of DXA in estimating %FM, FM and FFM changes is presented in figure 2. All the regression lines did not differ from the line of identity.

**Figure 3:**
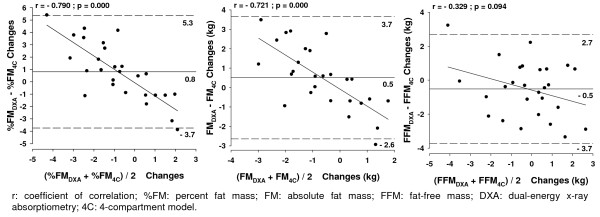
**Bland-Altman analysis of the agreement between methods in tracking body composition**. Bland-Altman analysis of the agreement between methods in tracking body composition changes (calculated as prior to competition minus period of stability). The middle solid line represents the mean differences between: %FM from DXA with %FM from the 4C model, the absolute FM from DXA with absolute FM from 4C model and absolute FFM from DXA with FFM from the 4C model. The upper and lower dashed line represents ± 2 SD from the mean i.e. 95% limits of agreement (± 1.96 SD). Trend line represents the association between the differences of the methods (DXA minus 4C model) and the mean of both methods in assessing %FM, FM and FFM. A significant correlation was observed for %FM and FM differences and the mean of the methods (p < 0.05) but not for FFM changes, as indicated by a non-significant p-value (p > 0.05).

The agreement between the 4C model and DXA is shown in figure 3. The 95% limits of agreement ranged from -3.7 to 5.3 for %FM, from -2.6 to 3.7 for FM, and from -3.7 to 2.7 for FFM. For %FM and FM changes, a significant trend was found between the difference of DXA and the 4C model with the mean of the methods (p < 0.01), except in detecting FFM changes (p > 0.05). Bland Altman plots of %FM, FM and FFM changes are shown in Figure 3.

Bland Altman analysis indicated that DXA tended to overestimate %FM and FM losses and to underestimate %FM and FM gains. The individual error reached ~9% of %FM and 6.3 kg of FM changes.

Although no relation was found between the differences of the methods and the mean of both methods in tracking FFM, the individual error reached 6.4 kg.

The performance of DXA in estimating %FM, FM and FFM comparing to the reference model both at the period of stability and prior to competition is presented in table 3.

**Table 3:** DXA Cross-sectional performance criteria* at the period of stability and prior to competition

	*Slope*	*Intercept*	*r*	*s.e.e.*	*Agreement*		
					
					*Bias*	*Limits*	*Trend*
%FM_Period of Stability_	1.03^b^	-3.24^a^	0.78	2.63	2.9	7.9, -2.2	-0.26

%FM_Prior to Competition_	0.97^b^	-3.38^a^	0.72	2.65	3.7	8.8, -1.4	-0.40

FM_Period of Stability_	0.95^b^	-1.57^a^	0.82	1.94	2.1	5.8, -1.7	-0.46

FM_Prior to Competition_	0.94^b^	-2.09^a^	0.78	1.89	2.6	6.2, -1.1	-0.29

FFM_Period of Stability_	1.09^b^	-3.14^a^	0.96	1.88	-2.7	1.1, -6.4	-0.42

FFM_Prior to Competition_	1.00^b^	3.51^a^	0.95	1.98	-3.2	0.6, -7.0	-0.15

Abbreviations: %FM: relative fat mass; FM: fat mass; FFM: fat-free mass;

*Performance criteria: slope, intercept, coefficient of correlation (r), standard error of estimation (s.e.e.) and the agreement (bias, limits and trend) between %FM, FM and FFM from the reference method and DXA

^a^Significantly different from 0, p < 0.05.

^b^Significantly different from 1, p < 0.05.

We further explored if the differences between methods were related with changes in body weight and FFM_D _and composition. Changes in FFM_D_, protein and mineral fraction were negatively associated with the difference of the methods in tracking %FM and FM while FFM hydration was positively related (p < 0.05). The FFM_D _and mineral were also positively related with the difference of the methods in assessing FFM changes thought a negative relation was observed (p < 0.05). Body weight changes were not significant related with the differences of the methods (p > 0.05), meaning that the differences between DXA and the 4C model in tracking %FM, FM, and FFM were not dependent on the magnitude of the weight changes.

Additionally, no association was found between absolute trunk, arms and legs FM and LST changes, obtained by DXA, and the differences of absolute FM, %FM and FFM changes obtained by DXA against the reference method (p > 0.05). Therefore, the differences between DXA and the 4C model in tracking absolute FM, %FM and FFM are not dependent on the magnitude of the trunk, arms or legs absolute FM and LST changes.

## Discussion

To our knowledge this is the first study to address the validity of body composition changes assessed by DXA in elite athletes using a 4C model. Dual-energy x-ray absorptiometry has been validated against criterion methods, such as multi-compartment models, for cross-sectional assessment of FM and FFM in athletes [12,28]. In our sample, the cross-sectional data indicated that the methods were highly related but, at an individual level, large limits of agreement for FM and %FM were found. Better results were found in estimating FFM on a group basis (r > ~0.95, SEE <1.98) and smaller limits of agreement. Both at the period of stability and prior to competition, significantly different results were observed in %FM, FM and FFM comparing to the reference method.

Few studies have validated DXA in tracking body composition [29-33], but none were performed with an athletic population. Accuracy was examined in the prediction methods in order to understand how DXA performed for the group as well as for the individual subjects in tracking %FM, FM and FFM. At a group level, prediction would be deemed accurate if 1) the predicted changes in %FM, FM and FFM from DXA were not significantly different from 4C model, 2) the regression between changes in %FM, FM and FFM from DXA had a slope not significantly different than one and intercept not significantly different from zero, and 3) the predicted changes in %FM, FM and FFM from DXA were highly correlated to 4C model. Using this approach: 1) no significant differences were observed between mean values, 2) all the study variables from DXA had regression lines with slopes not significantly different than one and intercepts not significantly different from zero, and 3) the Pearson correlation coefficients ranged from r = 0.53 to 0.62. The standard error of estimation (SEE) in the regressions ranged from 1.62% (FM) to 2.33% (FFM) meaning that small changes in body composition might not be accurately detected from DXA. For example: an athlete that gains about 5%FM the estimation error can be 5+1.62% or 5-1.62%, this represents an error of about 50% in the %FM changes in this athlete. The last item indicates that methods though related, the coefficients were not highly related (r < 0.80). Therefore, at a group level DXA did not present an expected accuracy.

At an individual level we examined the magnitude of the difference between DXA and 4C in tracking %FM, FM and FFM according to the Bland and Altman method [27]. Our results showed that, for an individual, there were large differences as indicated by the wide limits of agreement for each variable. Furthermore, a significant trend between the mean changes observed between DXA and 4C model and the difference between the methods was found for FM and %FM. These analyses suggest that individual estimates of changes in body composition when taken alone should be interpreted with caution, especially when tracking %FM and FM. DXA tended to overestimate %FM and FM losses and to underestimate the gains. The large limits of agreement in our study could cause an individual error of 9% FM, 6.3 kg of FM and 6.4 kg of FFM changes. In an athlete that changes FM, DXA can underestimate %FM changes by 3.7% and overestimate changes by 5.3%, given the limits of agreement (5.3, -3.7).

Other studies developed with non-athletic populations showed similar results in the accuracy of DXA in detecting small changes in %FM, though, contrary, FM reductions were underestimated [29-32]. In contrast, Houtkooper and colleagues [33] observed that DXA was a sensitive method for assessing small changes in body competition in a sample of postmenopausal women. In our study we observed small changes both in FM and %FM using the criterion model, but when using DXA significant differences were found only in FM from the period of stability to prior to competition. Neither DXA nor 4C demonstrated significant differences between FFM at the period of stability and prior to competition.

Schoeller et al. [34] demonstrated that DXA estimation for lean and fat mass can significantly vary among differing models (hardware and software) under different laboratory conditions. Based on 10 subjects the CV of DXA in our laboratory for FM is 2.9%. Given the magnitude of DXA error, small changes in FM might not be detected by the equipment. This information means that some of the changes that were observed in FM (somewhere between -2.9 and +2.9%) may not be accurately assessed as the values are within the precision of the equipment. Therefore, we performed additional analysis (data not shown) excluding cases where athlete's %FM changes were between -2.9% and 2.9% and repeated the regressions analysis. Even performing this step, the power of DXA in explaining changes in body composition remained poor.

Moreover we explored potential reasons for the large individual error in body composition assessment in DXA. Both at the period of stability and prior to competition significant deviations were observed between FFM components and the values obtained from chemical cadaver analyses [7]. Although no changes in FFM components were observed from the period of stability to prior to competition, an association between changes in the mineral fraction, hydration of FFM and protein fraction with the difference of the methods in assessing changes in FM and %FM. Also we found a relation between the difference of the methods in assessing FFM changes and FFM mineral fraction hydration. Both fat and LST have characteristic mass attenuation coefficients (R-values), assumed to be constant and different [35,36]. Previous studies reported R-values for fat and LST [18,37] but there is a lack of *in vivo *body elemental composition studies to understand if changes in FFM components would result in a R-value significantly different than the assumed by the equipment for general population. Any change in the assumed constant lean R-value would lead to soft tissue composition estimation errors [36]. Normal FFM hydration values may be critical in DXA [37,38]. However other studies pointed out that errors due to FFM hydration changes do not significantly affect FFM estimations [18,36,39]. In our study a positive relation was observed in FFM hydration changes and the difference between the methods in assessing FM and %FM, meaning that DXA tends to overestimate FM and %FM when there are gains in FFM hydration and to underestimate when there are losses in that FFM component. In estimating FFM an opposite trend was observed, resulting in DXA overestimating FFM when FFM hydration decreased and underestimate it when gains occurred. As an example, for an athlete who gained ~2.3% of the initial hydration of FFM, DXA would overestimate FM in ~3.5 kg. Theoretically changes in soft tissue hydration would result in alteration on lean tissue elemental proportions and R-value. Experiments suggest small errors in estimating fat with hydration changes [36]. Our findings might be attributed to the fact that, based on x-ray attenuation properties, water will appear to have a fat content of approximately 8% [37,38].

The degree of change in the amount of fat resulting from weight changes differs among specific regions of the body [6] and it is important to understand if changes in different body regions affects the accuracy of the equipment. In our sample we did not verify a relation between %FM, FM and FFM in trunk, arms or legs with the differences of the methods.

Another DXA assumption is related to the area of the body analyzed since DXA can only measure two compartments at one time [35,36]. First, DXA allows separation of the body into two compartments: bone mineral (Mo) and soft tissue (ST) [18]. Soft tissue can be separated into two molecular compartments: fat and LST [26]. This means that in pixels with bone, ST is not separately analyzed and the equipment assumes the fat content of the adjacent area analyzed [18]. Normally, 40% to 45% of the whole body scan contain bone in addition to ST thus, a systematic individual error is introduced as there might be variations in body composition between measured and non measured areas [35]. For example, the influence of arm and thorax on body composition estimation can be underrepresented due to the relatively large areas of bone in those regions [40]. This source of systematic error can be increased when tracking body composition compartments [41].

There may be systematic errors in comparing DXA estimates with those from another reference method. This can be attributable to inaccuracy of the reference method, inaccuracies on the DXA estimates, or a combination of the two [35]. We reviewed DXA considerations in trying to explain the inaccuracy observed in tracking %FM, FM and FFM in Judo athletes but it is also important to note that the 4C criterion model is not error free [28]. On a molecular level (2C) body weight can be divided in FM and FFM [42]. The 4C model [13] is based on the division of the body in 4-compartments at the molecular level: FM, water, mineral and protein. In this model FM was considered only as the fat lipids (nonessential) [26], as they are assumed to account for 99% of the ether-extractable lipids [43]. But, lipids can be divided into fat (nonessential) and non-fat (essential), to distribution, function, and solubility characteristics [43]. Non-fat lipids are then included in the FFM component [26]. If we consider the 70 kg Reference Man [44] the nonessential lipids correspond to 17% of body mass and the essential to 2.1%. Our judo athletes had mean values of ~9.2% and ~8.0%FM at the period of stability and prior to competition, respectively, which corresponds to approximately half of the %FM reported in the reference man. Given that the essential fat is relatively constant, it will represent a larger percent of total lipids in athletes. Also, regarding that some of our athletes presented values of ~3%FM, non-essential lipids is representing almost the same portions as the essential lipids.

This means that with the 4C criterion model we might be underestimating FM by taking into account a considerable portion of lipids as being part of the FFM component, which might question the use of this model to validate DXA in tracking body composition in extremely low fat level athletes.

## Conclusions

In conclusion, no significant differences were found between DXA and 4C model in estimating changes in body composition. However, unexpected inaccuracies were found using DXA for tracking adiposity, at a group level, mostly due to its poor ability in predicting body composition changes from the reference method as the methods were not highly related. Furthermore, given the large individual error DXA may not be accurate for detecting small physiological changes in athletes that need to achieve a target body weight prior to a competition.

## Competing interests

The authors declare that they have no competing interests.

## Authors' contributions

All authors read and approved the final manuscript. DAS: responsible for data analysis and manuscript writing; AMS responsible for data screening and collection and manuscript writing and supervision; CNM: responsible for data collection and pooling; and DAF, SBH and LBS: responsible for providing administrative support, supervision, and advice.
